# Material requirements planning with a novel lot sizing method and a new algorithm for production scheduling

**DOI:** 10.1038/s41598-025-13197-8

**Published:** 2025-07-29

**Authors:** Seyed Mahdi Javadi, Seyed Jafar Sadjadi, Ebrahim Teimoury, Ahmad Makui

**Affiliations:** Iran University of Science and Technology, Tehran, Iran

**Keywords:** Material requirements planning, Inventory control system, Bill of materials, Master production schedule, Lead time, Lot sizing, Supply chain management, Engineering, Mechanical engineering

## Abstract

Material Requirements Planning is an intelligent information system and a decision maker that determines the type of raw materials, the quantity of them, and their supplying time in order to realize production schedule of products and work-in-process items in a manufacturing system. In each manufacturing system, if we split production costs into material, labor, and overhead costs, we will observe that over 60% of total production costs are related to material. Therefore, it is necessary to consider an appropriate inventory control system to manage the costs of inventory system to provide right inventory, with right quantity, at the right time, at the right price, and with appropriate quality. Lot sizing methods in previous researches do not provide feasible and operational solutions, or have parameters such as holding costs and set up costs that are not identified in financial accounts. To solve this research gap, in this paper, we presented a new lot sizing method considering two important parameters including batch size of the items and the minimum order quantity that a supplier provides for the items. Simultaneously, we designed a new production scheduling algorithm in Master Production Schedule component for a manufacturing system with *k* production lines and *m* products with *n* Bill of Materials for each product. At the end, we programmed our new mentioned contributions and developed Material Requirements Planning module of a Supply Chain Management system.

## Introduction

**Fig. 1:**
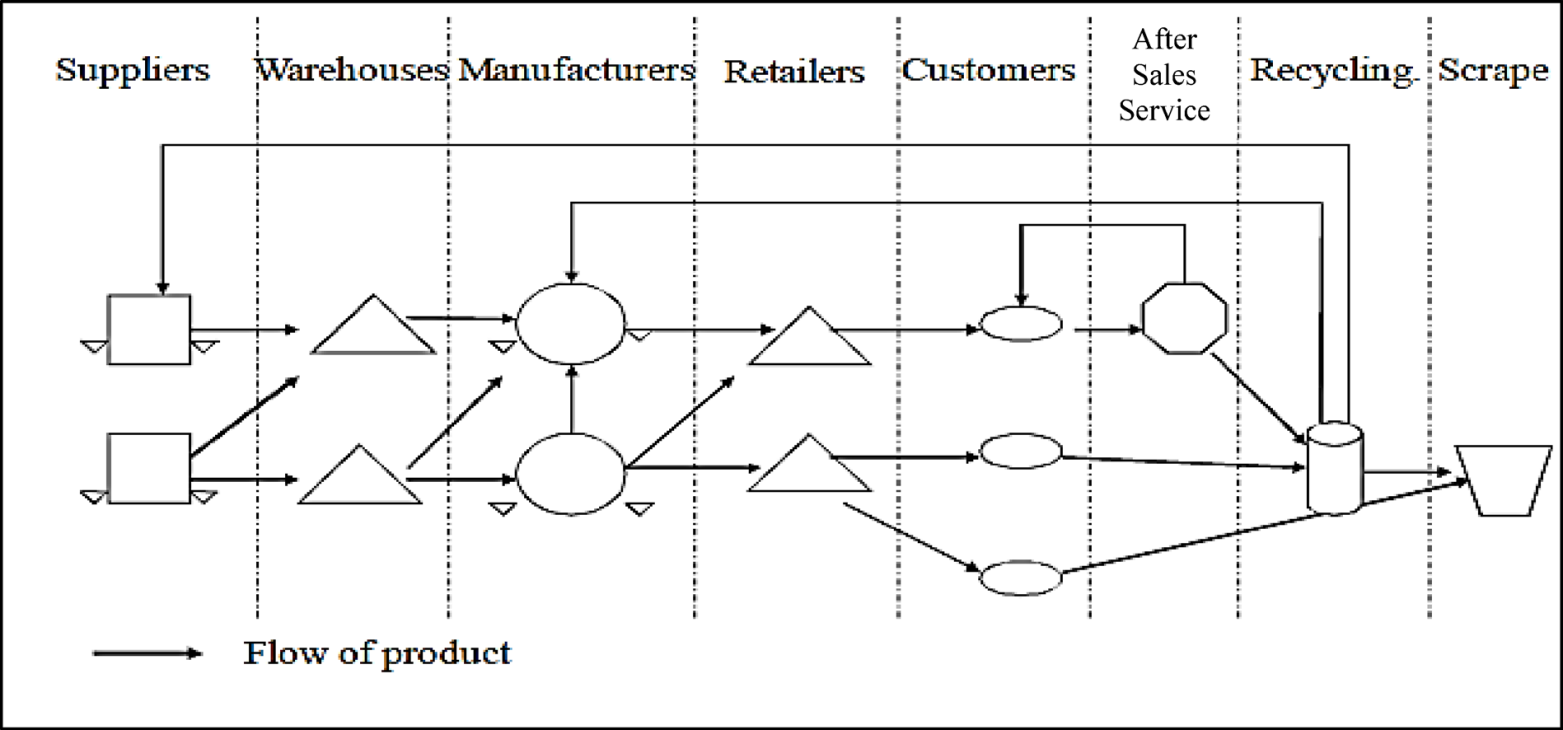
Supply chain components.

Supply chain is a network that includes three stages of purchasing raw materials from factories and suppliers, performing operations on raw materials and producing products, and distributing these products to customers. The components of these steps are shown in Fig. 1. Supply chains are seen both in the industry and production sector and in the service sector, and the complexity of the chain can be different from each economic enterprise to another enterprise^1,2^. There are many tools available to manage supply and demand and maintain market balance to increase chain profits. For example, it is possible to influence the supply level by determining the optimal transportation and distribution system, changing the production capacity and determining different inventory levels, and by applying marketing and advertising principles, the demand level can be changed.

Among all available tools, the inventory control system plays an important role to increase the profitability of the supply chain, maintain and expand the customer network, and improve the organization. A proper inventory control system can respond appropriately to system demands and ultimately affect revenue. In fact, the implementation of a suitable inventory control system is the main and key pillar for organizations that purchase raw materials, produce their product(s) and distribute for sale in the market.

If we divide the production costs into three kinds of raw materials, labor and overhead costs in any production system, it can be observed that over 60% of the total costs are related to the raw materials cost. Therefore, in every production system, it is necessary to consider a suitable inventory control system to manage the inventory system costs to provide the appropriate inventory, with the right quantity, at the right time, at the right price, and with the right quality^1–3^.

One of the types of inventory control systems is Material Requirements Planning (MRP), which is actually an intelligent information and decision-making system that determines the type of raw materials, their quantity, and their supply time in order to realize the production plan of products and Work-in-Process (WIP) items in a production system. The inputs of this model include Bill of Materials (BOM), Master Production Plan (MPS), lead time, and inventory file. The outputs of this model are the raw material supply plan as the main output and its sub-outputs include control of consumables items of production plan, financing planning, budgeting, etc.

The objective and contribution of this research is the correct implementation of the MRP system through our new lot sizing method. Existing MRP model with current lot sizing methods cannot run in real environment because there are some gaps between outputs of MRP and constraints of suppliers to supply the items. For a raw material like citric acid, if we run the MRP for an MPS, suppose that the required amount of this product will be 8 kg, but the supplier cannot supply this amount because the packaging of this product may be 50 kg or the supplier may not provide less than 1000 kg of it. To fix these gaps, a new production scheduling algorithm will be introduced for a production system with *k* production lines and *m* products with *n* BOM. After that, a new lot sizing method will be presented and MRP system will be developed.

## Literature review

In this section, according to the studies that have been done from various sources regarding the previous research in this field, the development course of MRP from the beginning to its expansion and various developments will be briefly reviewed.

In 1966, at the American Production and Inventory Control Society (APICS) conference, the first research on material requirements planning was initiated by Joe Orlicky at IBM, Oliver Wight and George Plossl at Stanley Works, and at the 14th APICS conference in 1971, Orlicky and his colleagues in an article entitled “MRP, a hope for the future or the present reality - a case study”, they claimed that MRP is far more efficient than point-of-order (ROP) methods. In the 1980s, Oliver Wight proposed the need to integrate production with the planning and control of other production-related resources such as finance and distribution and invented manufacturing resource planning (MRPII)^4^.

In a research, Aziz Hamid and his colleagues reported on the use of MRP by 22.7% of Malaysian companies^5^. In an article, Vincent Mabert reviewed the developments and developments of MRP during the years of its formation^4^. The implementation of MRP in a manufacturing company in Greece was investigated by Manthou and his colleagues, and the amount of use of this inventory control system in the relevant company, the problems they face and the benefits of its implementation were explained^6^. The effect of perishable inventory in the decision making of the cumulative determination method in the MRP system was researched by Johnny C. Ho and his colleagues^7^. Fuzzy MRP model considering total fuzzy cost, fuzzy available capacity, and fuzzy demand was developed by Mula^8^. In another study, an algorithm using the branch-and-cut approach to determine the backlog in the MRP system was presented by Mirmohammadi and his colleagues^9^. Continuous MRP model with zero confidence inventory and cumulative determination by L4L method was proposed by Sadeghian^10^. In an article, Diaz-Madronero and his colleagues presented the MRP model with fuzzy lead time with a fuzzy multi-objective integer programming approach^11^. In a research, Cornelis de Man and his colleagues addressed the issue of why Excel software still plays an important role in MRP and production planning and found that this issue is due to the dynamic nature of the MRP model^12^. In another research, a hybrid algorithm of genetic algorithm and forbidden search for MRP system with limited capacity was presented by Sukkerd and his colleague^13^. A technique for planning material requirements and production planning was introduced by Segerstedt^14^. A 50-year review of Material Requirements Planning model achievements was interpreted by Bogotaj^15^. In Ramya et al.‘s article, an enterprise software framework for the integration of material requirements planning with workshop scheduling has been developed^16^. In a research, a mixed model of limited chance planning has been developed to solve the MRP problem with mixed uncertainty by Zhu and his colleagues, in which there are both randomness and fuzzy discussions in the decision-making process regarding the determination of the stockpile size. In fact, the purpose of this model is to determine the accumulated size of all items despite random demand and fuzzy capacity limitation^17^. A model examining how a firm might select the package size and price for a product that deteriorates over time has been presented^18^. The application of simulation-based optimization for material requirements planning has been illustrated on a high-dimensional real world use case^19^.

As can be seen in the literature review, there is not a lot sizing method to consider batch size and minimum order quantity for items of supplier in MRP model. Thus, our novel lot sizing method and its prerequisites (including our new production scheduling algorithm) for designing MRP module will be presented in the next section.

## Modeling and algorithms

**Fig. 2:**
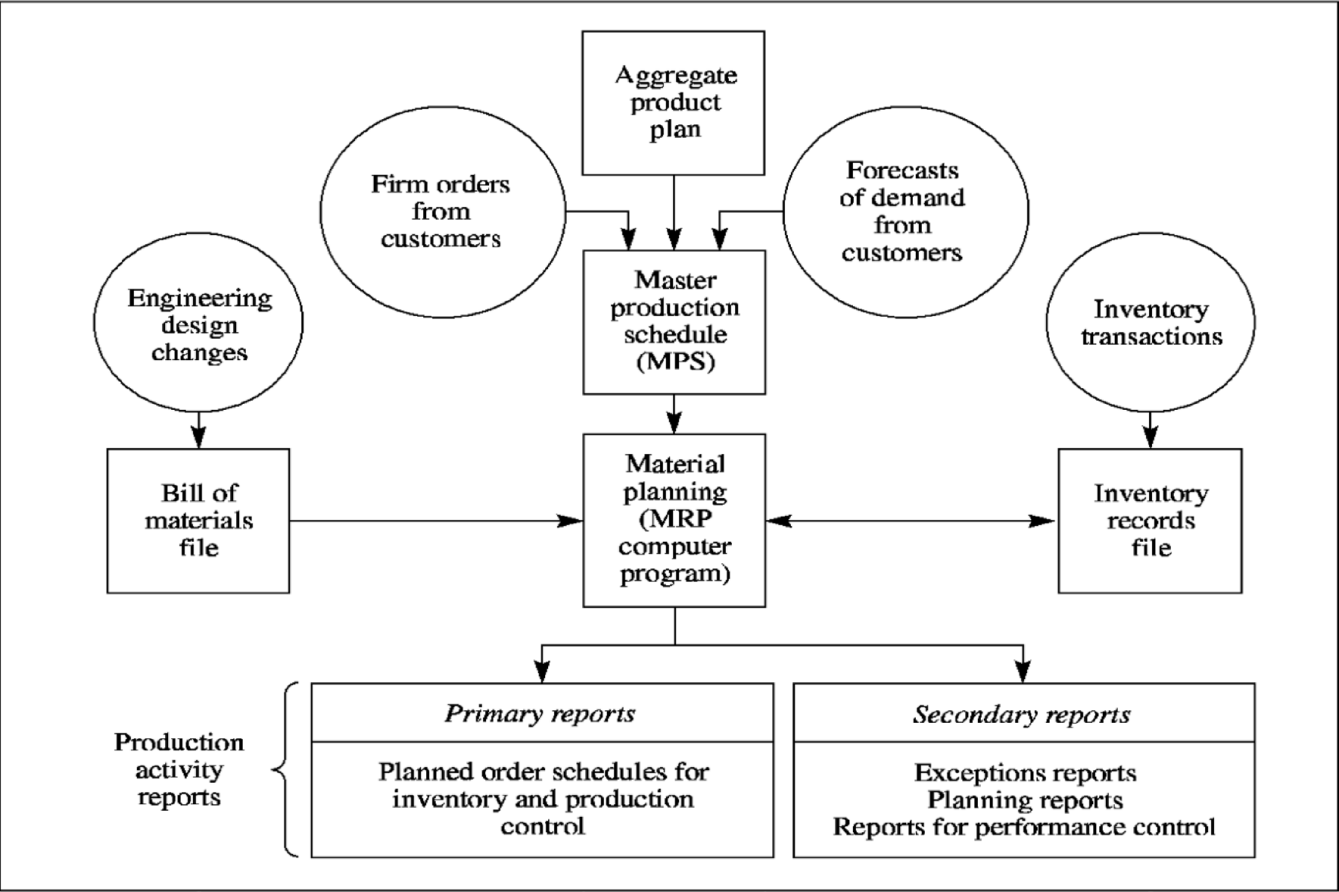
MRP model components.

In order to implement the MRP model shown in Fig. 2 in according to the requirements and managerial insights presented in^3^, and based on our new lot sizing method, the building theory along with case study were employed. Also, the agile management framework (Scrum) was used and new algorithms were designed in each part of the system. Then it was programmed using Visual Basic (VB) language and UserForms and related reports were designed and tested. It will be assumed that lead-times, BOM coefficients, and demand are deterministic.

The MRP system has a module and this module consists of several components and a special algorithm is designed for each component.

### Production calendar creation algorithm

As mentioned in the previous sections, one of the important inputs of the MRP model is the master production plan or MPS, which itself also has important inputs, including the production calendar. In fact, for every production that is going to be done in a production system, a production calendar is required in which the following items are specified:

- When will be the start date and end date of production?
- How many operators are available on each day of this calendar?
- Is overtime allowed on holidays or not?
- How many days and weeks of the year does each date in this calendar correspond to?

Due to the fact that the production planning period may be monthly or more than one month and the production calendar records will be numerous, therefore, it is necessary to design an algorithm to create a production calendar. For this purpose, the pseudocode of the production calendar creation algorithm is given in Table 1. In this algorithm, first the start date and the end date of the production calendar are taken from the user, then it is controlled that the end date of production planning is greater than the start date. If the start and end dates of the production calendar are correct, the difference between these two dates determines the number of days in the calendar. Then, in a loop, one day is added to the calendar start date according to the number of days in each iteration, and in each iteration, the date, days of the week, days of the week code, the number of days of the year, the number of weeks of the year, financial year, the number of available human resources, holidays The official calendar, and the status of overtime allowed during holidays, is specified and saved as a production calendar document. Each time this template is called, a new production calendar can be created for MPS input.

**Table 1:**
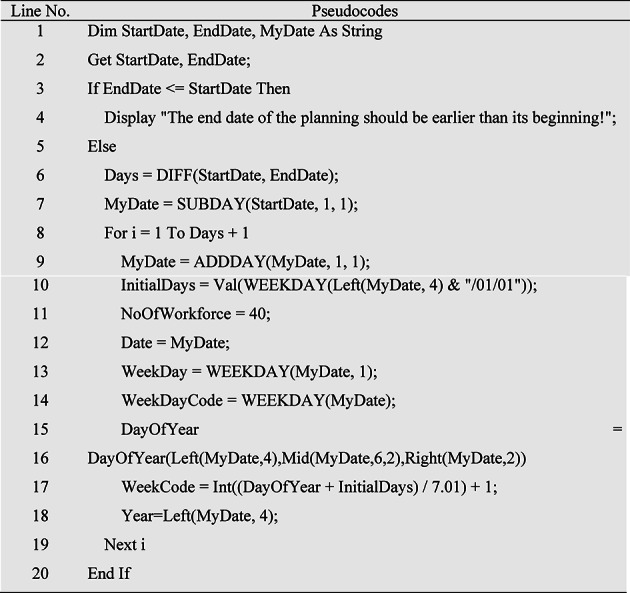
Pseudocode of the algorithm for creating the production calendar.

**Fig. 3:**
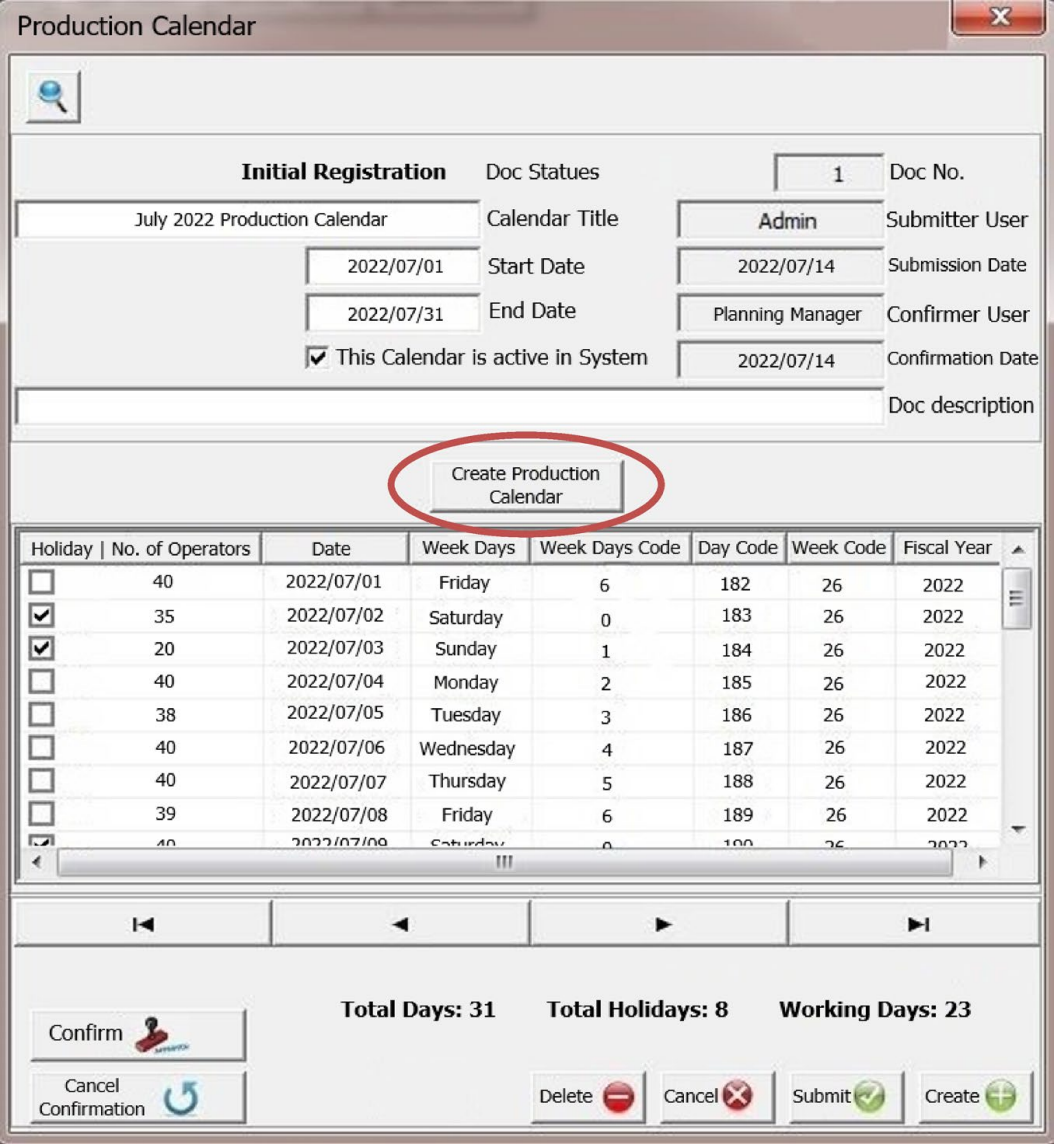
Production calendar.

This algorithm has been ran for a start and end dates and the output of production calendar has been illustrated in Fig. 3.

### Production scheduling algorithm

After determining the inputs of MPS (such as the production calendar, sales plan or budget related to the production calendar, production lines, standard production capacity, number of standard production workforce, and so on), it is time to tune the parameters of the products and semi-finished products in the MPS (such as the code of product formulation or BOM, the duration of the work shift, and the production quantity). Finally, a decision should be made regarding to the produce or purchase semi-finished products. After this stage, it is necessary to schedule the production of products and semi-finished products in MPS. For this purpose, the pseudocode of the production scheduling algorithm is given in Table 2. At the beginning of this algorithm, the feasibility of MPS is checked, assuming that the time required to produce products and semi-finished products related to a production line does not exceed the nominal capacity of that production line. Otherwise, the MPS parameters must be adjusted again to finally make MPS possible. In the following, the products in MPS, which are arranged according to the production priority, are checked, which production line each one belongs to, and the number of production days required in the production line corresponding to that product and on the corresponding day in the production calendar, as well as the force limit. human will be considered on that day and if that day is not a holiday or overtime is allowed on that day, the corresponding product will be allocated to the corresponding production line and according to the number of days required for production on the corresponding day(s) and the number of manpower required each day will be deducted from the available manpower that day. Considering that each product or semi-finished product in MPS may require more than one day of production, therefore, at the end of the algorithm, the first production time of each product that is scheduled in the production calendar will be allocated to the corresponding product in MPS so that it should be used in MRP calculations to determine the supply time of the relevant product raw materials.

**Table 2:**
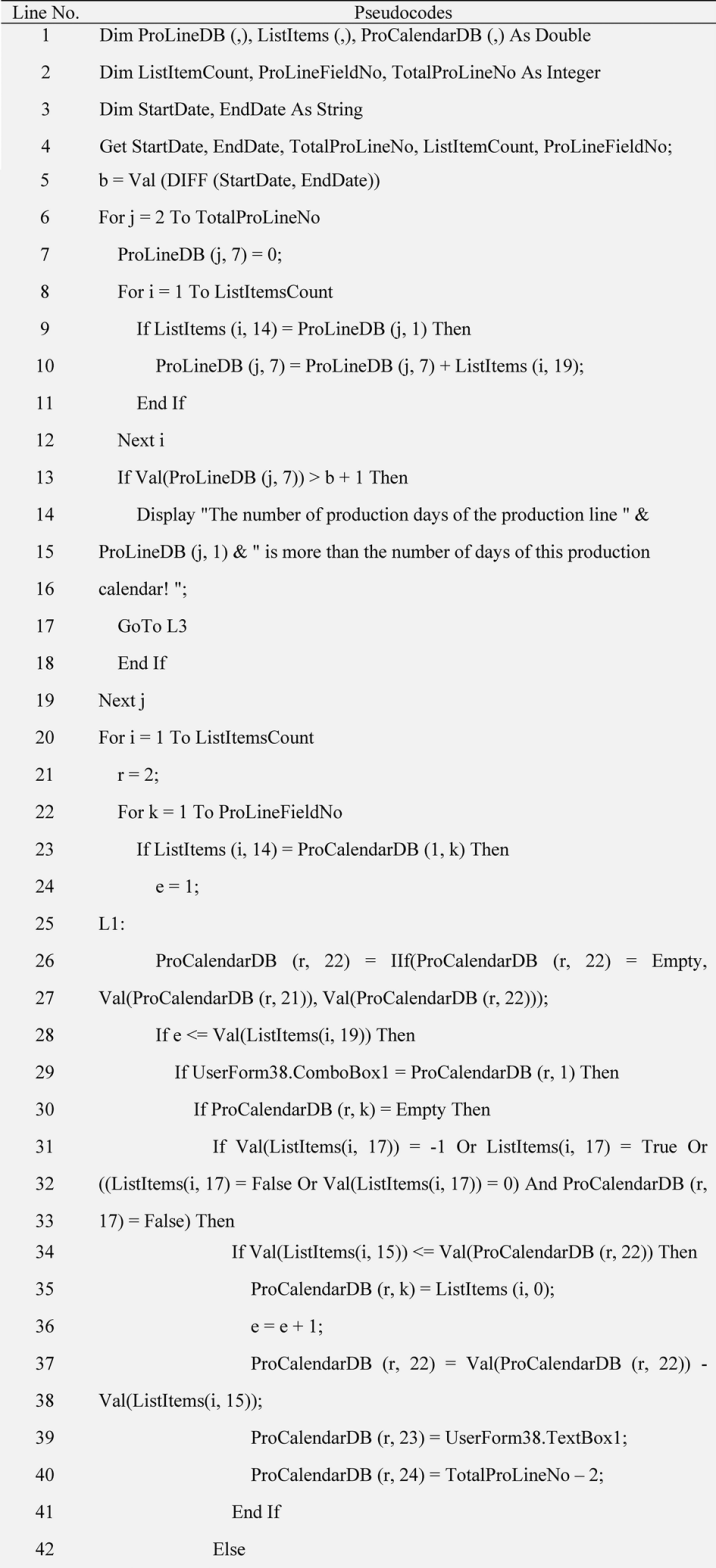

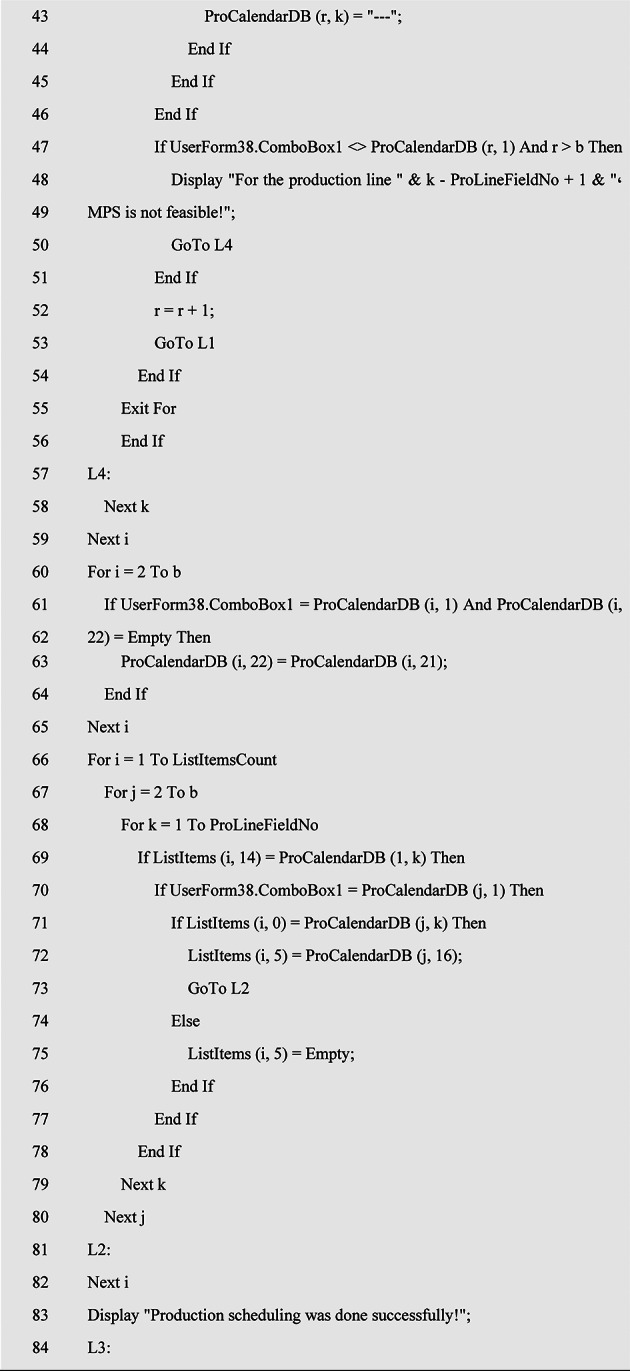
Pseudocode of the production scheduling algorithm in MPS with *n* products and *k* production line.

**Fig. 4:**
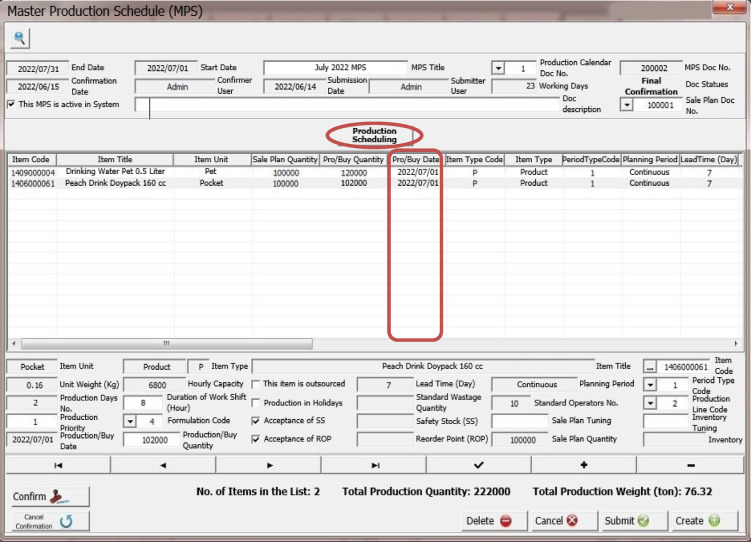
Production scheduling in MPS.

This algorithm has been ran for an example of a production calendar and the output of MPS has been illustrated in Fig. 4.

### MRP algorithm with a new lot sizing method

Due to the fact that the infrastructure for injecting MRP inputs into the model has been prepared in the previous sections, therefore, in this section, the MRP calculation algorithm and the determination of the accumulated size, taking into account the two limitations of the size of the packaging of the goods and the minimum order quantity of the suppliers’ goods, as described in Table 3 designed. For this purpose, at the beginning of the algorithm, it is controlled that the production program in question is selected so that MRP calculations are done for that MPS because there are separate MRP calculations for each MPS. Next, the quantity of production of each product and semi-finished product in MPS, according to the formulation intended for production, is multiplied by the raw material consumption factor in the corresponding BOM and the required quantity of raw materials will be obtained. Now, the required quantity will be deducted from the stock of the warehouse and from the quantity of the projected inventory of that product to obtain the purchase quantity. But the key point is that mostly the suppliers are not able to provide this quantity of items that is obtained at this stage, because the supply of goods is a function of the minimum order quantity that they provide and the size of the product packaging. Therefore, at this stage, if the purchase quantity is less than the minimum order quantity, it will be adjusted to this quantity, and if the purchase quantity is more than the supplier’s minimum order quantity, it should be rounded as a multiple of the product packaging size, until the multiple that is greater than the purchase quantity is the shortage. Raw materials do not happen and the production plan is realized. Their supply date is also obtained by deducting the supply time of each raw material from the date of the corresponding product production plan which will be obtained in the previous algorithm. In cases where a raw material is present in the BOM of several products, the earliest date of the production plan of the relevant products will be taken into account and will be deducted from the procurement time of that raw material until the supply date of this product is obtained and realized by the timely supply of the production plan. Therefore, this will be a new method to determine the accumulated size of goods, which, while this issue is not found in the literature, it will also be the most effective method from the point of view of operation, because as mentioned in the previous chapter, the methods in the literature are mainly based on the ordering cost parameters and The maintenance cost depends on the fact that these parameters are not defined in the financial system of production centers.

**Table 3:**
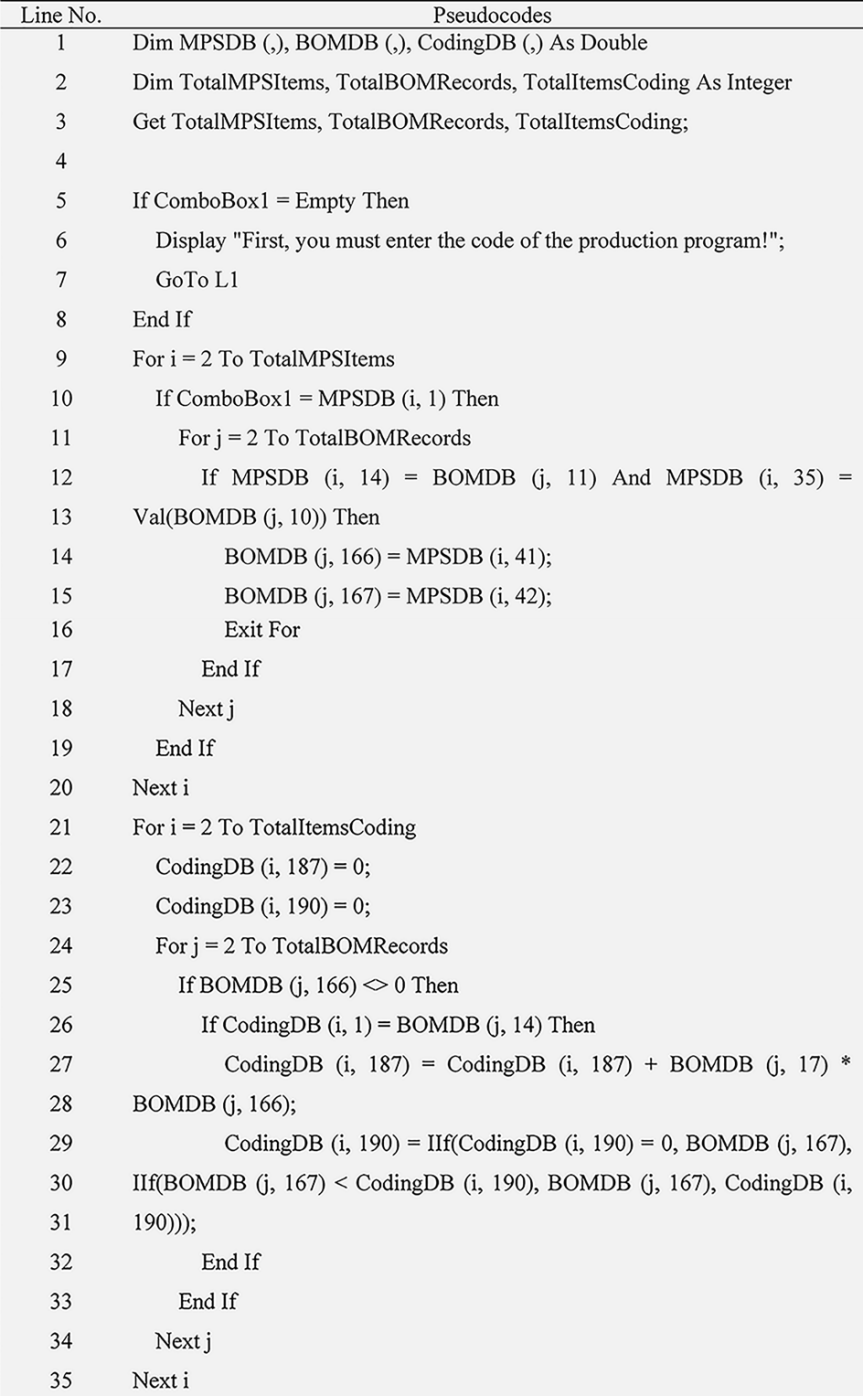

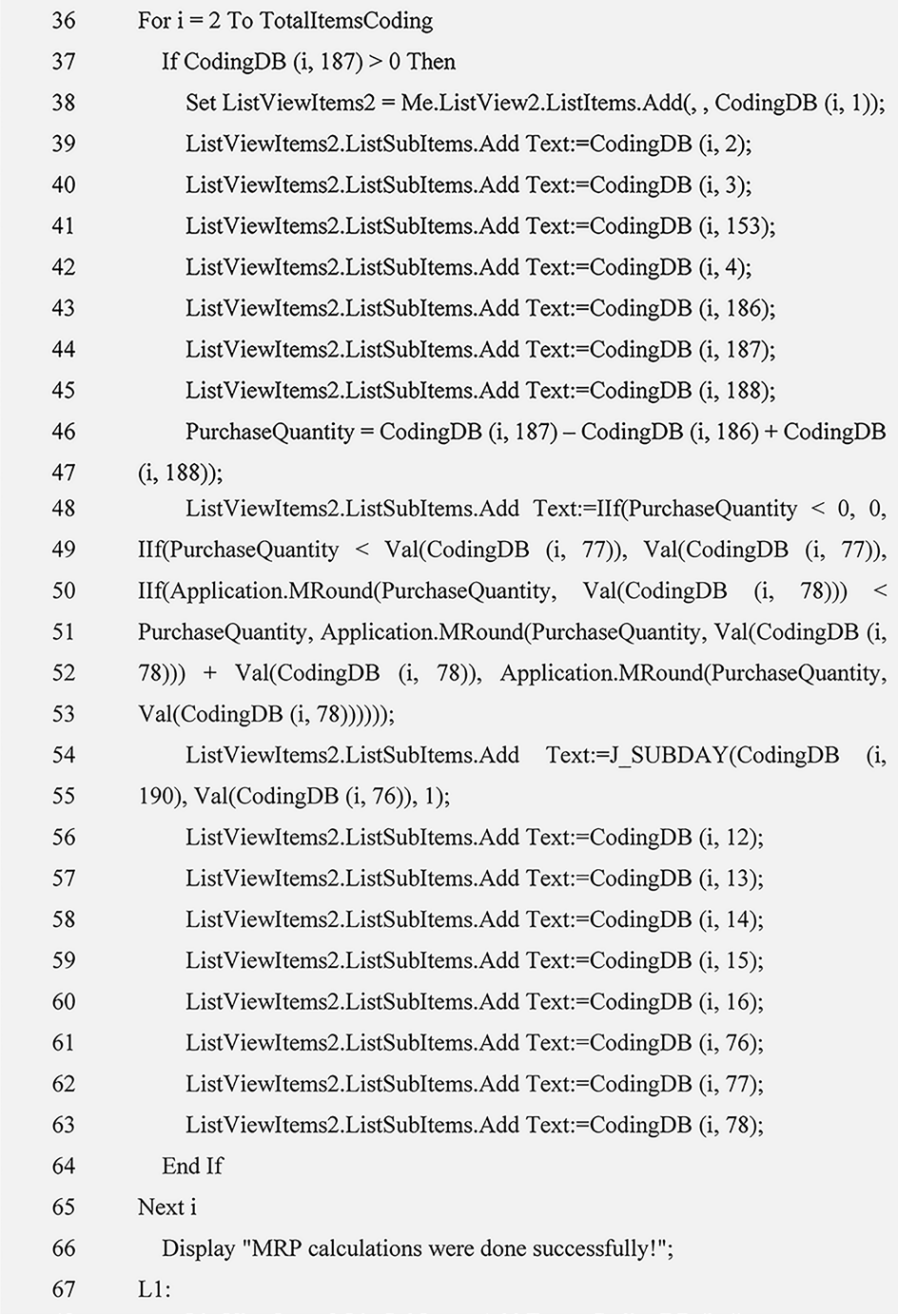
MRP calculation algorithm pseudocode.

**Fig. 5:**
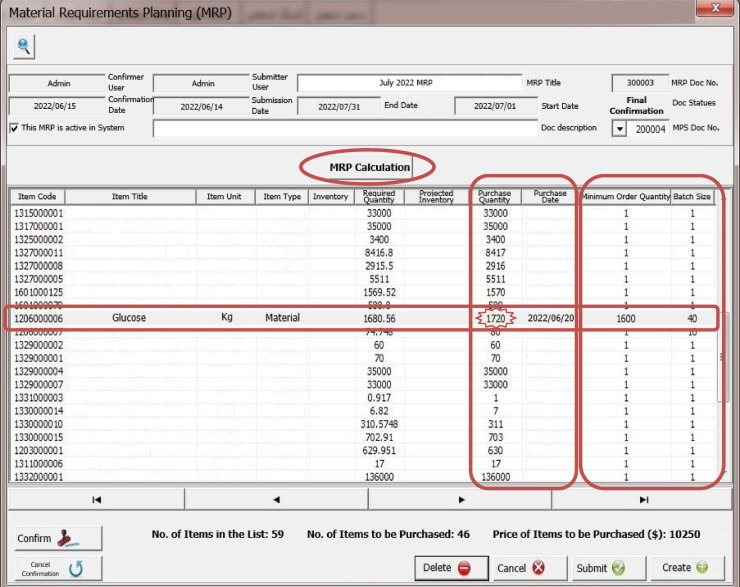
MRP calculation.

This algorithm has been ran for an example of a MPS and the output of MRP has been illustrated in Fig. 5.

## Discussion

**Fig. 6:**
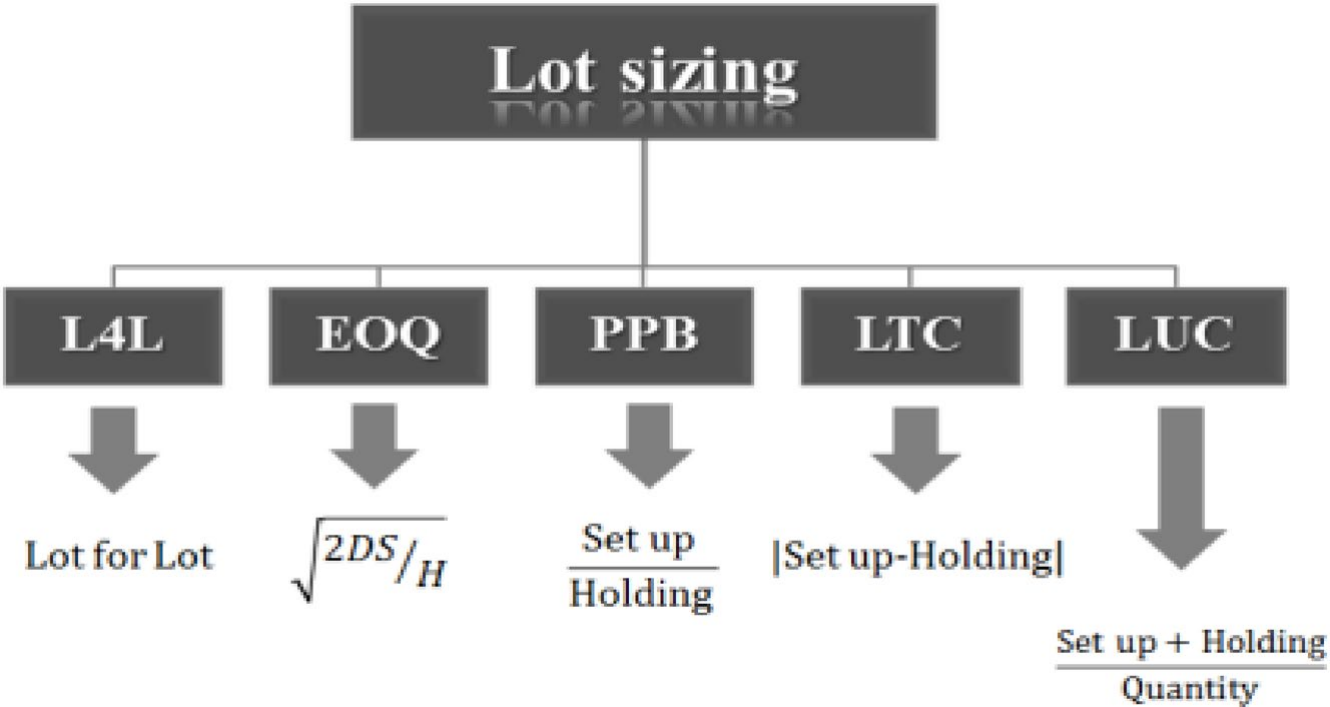
Lot sizing methods.

There are several lot sizing methods in the literature that has been illustrated in Fig. 6 with their formulation^3^ and we will review each of these methods in the following.

### Lot for lot

In the Lot for Lot method, also known as L4L for short, the amount of lot ordered from each raw material for suppliers is exactly equal to the multiplying of the product existing in the production plan in the technical coefficient of the raw material in the BOM of the product, which according to the lead time of the raw material will be provided.

### Economic order quantity

This method, also known as the economic order quantity (EOQ) model, states that each raw material is calculated from the following equation and ordered for suppliers:

1 $$\:Q=\sqrt{\raisebox{1ex}{$2DS$}\!\left/\:\!\raisebox{-1ex}{$H$}\right.}$$

Where D is the average demand for the desired raw material in a given period of time (which is obtained by multiplying the demand of the product in the technical coefficient of the raw material in the BOM of this product), S is the cost of each order, H is the holding cost of the unit item at a given time period, Q is the order quantity.

### Part period balancing

The part period balancing method (PPB) states that for each raw material, the net demand for each period is accumulated, and whenever the holding cost of them is close to the number in the following equation, the amount accumulated in one step will be ordered for suppliers:

2 $$\:Economic\:Part\:Period=EPP=\frac{Set\:up\:Cost}{Holding\:Cost}$$

### Least total cost

In the least total cost (LTC) method, the minimum cost is when the difference between holding cost and set up cost is reduced to a minimum. In this case, the required accumulation of time periods up to this minimum difference is aggregated for a raw material and ordered for suppliers.

### Least unit cost

In the method of least cost per unit of goods (LUC), the minimum cost is when the summation of holding cost and set up cost for per unit of raw material is reduced to a possible minimum quantity. In this method, the lot required for time periods up to this minimum is aggregated and ordered for suppliers.

Except L4L method, other methods depend on set up cost and holding cost for every raw material but these parameters cannot compute easily or does not exist in accounting systems in real environment. Also, L4L method is not compatible with real condition because for a raw material like citric acid, if we run the MRP for an MPS with L4L method, as an example, the required amount of this item will be 8 kg, while the supplier cannot supply this amount because the packaging of this product may be 50 kg or the supplier may not provide less than 1000 kg. So, it will be needed a lot sizing method to consider these constraints. The contributions of this study i.e. the presented algorithms in Sect. 3, are a package to solve this problem in a pragmatic way in real environment. Moreover, MRP module has been programmed and developed with presented algorithms in order to help mangers to make a right and careful decision for purchasing raw material in a feasible and optimal condition.

## Conclusion

Nowadays, due to the increasing dynamics of environmental and competitive conditions in the domestic and global markets, speed and accuracy in decision-making are very important for managers of organizations. In production organizations, making a decision on the supplying of raw materials for the realization of the production plan is one of the most important management decisions such that this issue can determine profit and loss of a firm in a financial year. Because if we divide the production costs of a manufacturing system into three parts including material cost, labor cost, and overhead cost, about 60–90% of the cost of operating revenue is related to the material cost^3,20^. Therefore, it is important for the mangers of a manufacturing system to select a proper inventory control system and deploy it in a correct and appropriate way. In the same direction, in addition to the algorithms that have been provided in previous sections, the management insights obtained from the experiences of the authors about implementation of MRP module have been documented so that it can be used by the readers. Unlike the methods available in the literature, our presented lot sizing method considering batch size of the items and the minimum order quantity that a supplier provides for the items, is an exact and feasible method in real and dynamic environment. So, the authors will suggest to related decision makers to employ our presented model in this research work to have an efficient and responsive inventory system and finally maximize their customer satisfaction. Some challenges including failure to accompany personnel in deployment of presented MRP module, trading commission in public companies for purchasing raw material, bottlenecks and stoppages of production lines, and fuzzy BOM can cause nervousness and disorders in MRP deployment. In order to solve these problems, some future researches are proposed for enthusiast in the following.

In fact, the lack of a system with industrial engineering and system thinking approaches that integrates different processes in an integrated system is a gap that is evident in the existing reality of a supply chain. Actually, the processes are designed as islands and are operated in a vacuum and finally the beneficiaries are not fully satisfied with the output of the work. Therefore, development of MRP module in this study and providing an integrated system of supply chain planning with a systemic approach and new algorithms can be researched to fill this gap.

Another topic that has been determined for future research is providing a material requirements planning system with fuzzy BOM of products. In some production systems, some raw materials and semi-finished products that are present in the BOM of a product do not necessarily have a constant consumption factor and distort the output of the material requirements planning model. For example, glucose, which is a raw material in the BOM of various products such as mayonnaise sauce, ketchup, etc., has a factor called Brix, which can change consumption coefficient in the BOM. In order to solve this research gap, according to the factors related to those raw materials, a fuzzy number can be assigned for its consumption coefficient and a new model can be presented in this regard.

Third topic that can be carried out to solve nervousness in MRP deployment including bottlenecks and stoppages of production lines is identification and fixing bottlenecks of production line by using a simulation approach^21^.

## Data Availability

The datasets used and/or analyzed during the current study available from the corresponding author on reasonable request.
